# Antenna Booster Element for Multiband Operation

**DOI:** 10.3390/s24092867

**Published:** 2024-04-30

**Authors:** Elena García, Aurora Andújar, Jaume Anguera

**Affiliations:** 1 Ignion, 08174 Barcelona, Spain; elena.garcia@ignion.io (E.G.); aurora.andujar@ignion.io (A.A.); 2 Research Group on Smart Society, La Salle Engineering, Universitat Ramon Llull, 08022 Barcelona, Spain

**Keywords:** wireless devices, multiband antenna, Internet of things (IoT), antenna design, antenna boosters, matching network

## Abstract

The escalating demand for versatile wireless devices has fostered the need to reduce the antenna footprint to support the integration of multiple new functionalities. This poses a significant challenge for the Internet of things (IoT) antenna designers tasked with creating antennas capable of supporting multiband operation within physical constraints. This work aims to address this challenge by focusing on the optimization of an antenna booster element to achieve multiband performance, accomplished through the design of a band-reject filter. This proposal entails a printed circuit board (PCB) measuring 142 mm × 60 mm, with a clearance area of 12 mm × 40 mm, incorporating an antenna booster element of 30 mm × 3 mm × 1 mm (0.07 λ). This configuration covers frequencies in the LFR (low-frequency range) from 698 MHz to 960 MHz and the HFR (high-frequency range) from 1710 MHz to 2690 MHz. A theoretical analysis is conducted to optimize bandwidth in both frequency regions. Finally, a prototype validates the analytic results.

## 1. Introduction

The rapid evolution of technology is fundamentally transforming our world, with the internet playing a leading role. The Internet of things (IoT), a network of interconnected devices collecting and sharing data, is a prime example of this transformation. These IoT systems rely on three key components: sensors for data acquisition, communication channels for data transmission, and a cloud network enabling device connectivity and data analysis [1,2,3,4].

The role of antennas is to function as the gateway for data transfer in IoT systems, forming the foundation for reliable communication between devices, sensors, and the central network across various applications like industrial automation and agricultural monitoring.

While traditional single-band antennas suffice for conventional equipment, contemporary systems demand more versatile and efficient solutions, such as dual-band or multiband antennas [5,6,7,8,9,10,11,12,13,14,15,16,17,18,19,20,21,22], to accommodate operations across multiple frequencies. Moreover, compact antenna form factors are crucial, considering the diverse sizes and internal electronics of these devices.

Historically, antenna design has leaned heavily on intricate geometries, depending on redesigns to resonate at specific frequencies for each platform [23,24,25,26,27,28,29,30,31,32]. This presents a notable challenge for antenna designers since iterative procedures are required to optimize the antenna geometry. In response, antenna boosters were developed to streamline the process. These elements are electrically small and have non-resonant impedance, typically at the lowest frequencies of operation, affording control over the frequency band of interest through the implementation of a matching network [33,34].

The aim of this research is to optimize the performance of an antenna booster element with dimensions of 30 mm × 3 mm × 1 mm, intended to operate from 698 MHz to 960 GHz and from 1710 MHz to 2690 MHz. The optimization focuses on enhancing the filter necessary for one of the antenna ports to achieve optimal functionality.

This paper is structured as follows: the antenna booster element is presented in Section 2; the design of the filter is described in Section 3 to optimize bandwidth at the low- and high-frequency regions; electromagnetic simulations are shown in Section 4 to evaluate the optimum filter; experimental validation is explained in Section 5; a discussion comparing other works is explained in Section 6; and finally, conclusions are drawn in Section 7.

## 2. The Antenna Booster Element

**Figure 1:**
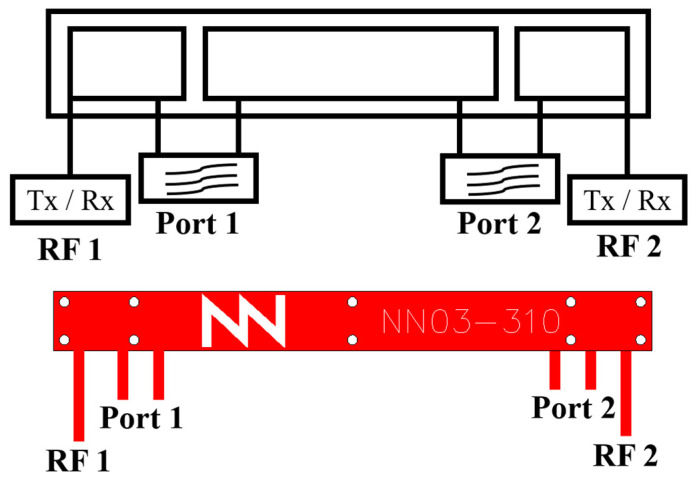
A 30 mm × 3 mm × 1 mm antenna booster element with four ports for multi-radio purposes.

The antenna booster element is a compact, non-resonant component fabricated on FR4 material with dimensions of 30 mm × 3 mm × 1 mm (height). Integrating a matching network in the RF port in use, composed of lumped components such as inductors and capacitors, allows the element to be tuned for the desired frequency band. This element has a total of four ports, including two radio-frequency (RF) outputs and two filter ports (Figure 1) [35].

**Figure 2:**
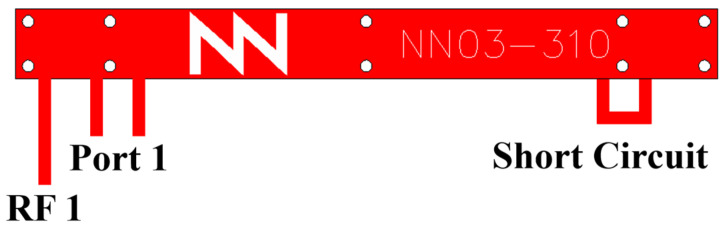
A 30 mm × 3 mm × 1 mm antenna booster element with 1 RF port and 1 filtering port; RF2 is unconnected and Port 2 is short-circuited.

RF1 and RF2 are designed to enable multi-radio applications. However, in typical single-radio scenarios, RF2 remains unused. In such cases, Port 2 can be either left open or short-circuited. As far as a single radio is concerned, Port 2 can be used for fine-tuning purposes. (Figure 2).

**Figure 3:**
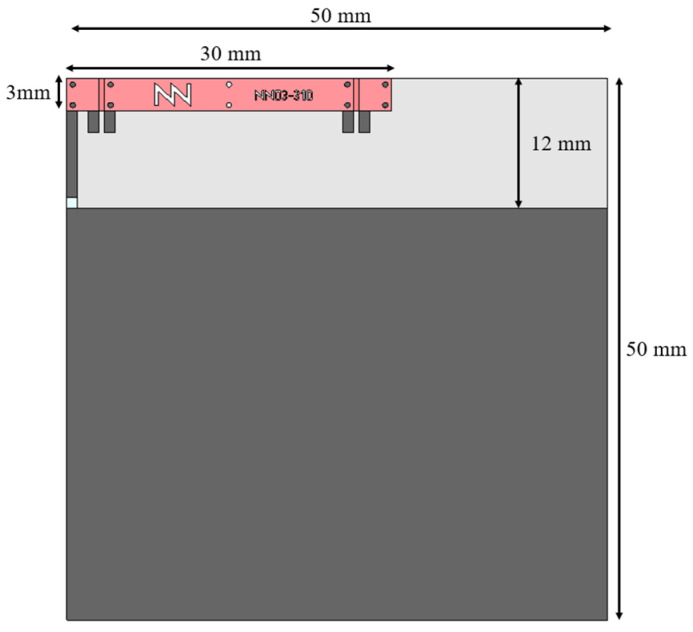
Simulated 50 mm by 50 mm PCB with FR4 substrate of 1 mm thick (ε_r_ = 4.15, tanδ = 0.02) with a 12 mm by 50 mm clearance area embedding a 30 mm × 3 mm × 1 mm (height) antenna booster element.

**Figure 4:**
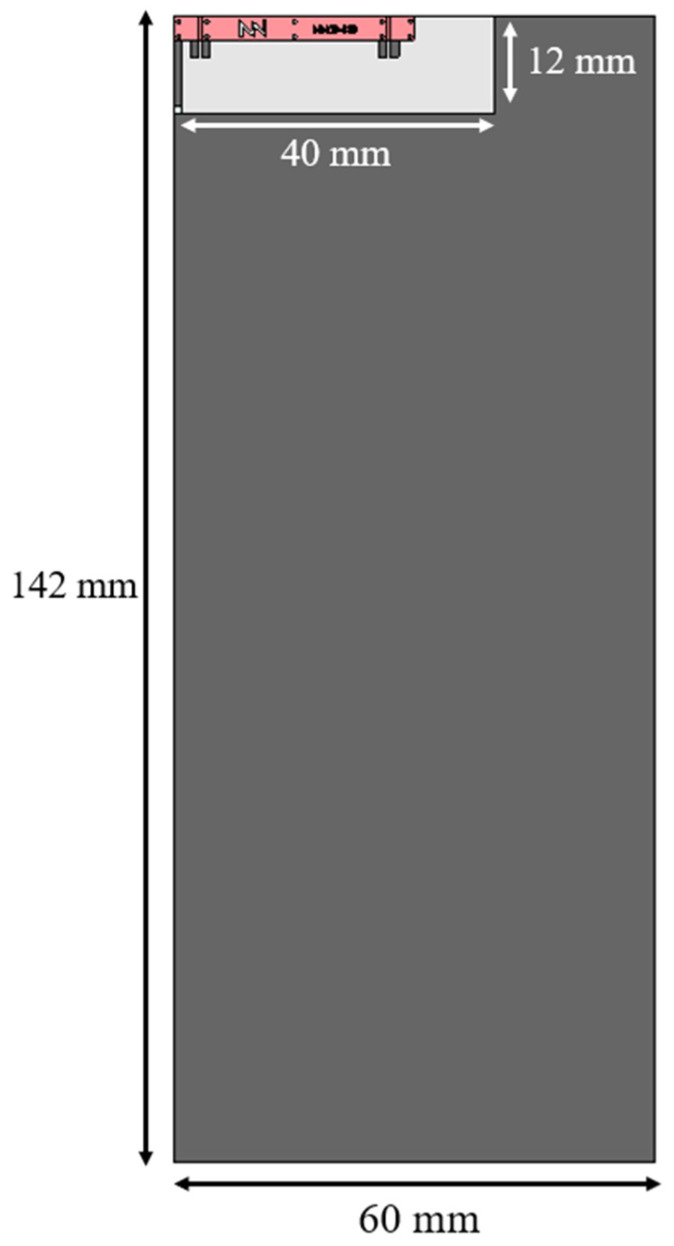
Simulated 142 mm by 60 mm PCB with FR4 substrate of 1 mm thick (ε_r_ = 4.15, tanδ = 0.02) with a 12 mm by 40 mm clearance area embedding a 30 mm × 3 mm × 1 mm (height) antenna booster element.

This section explores the optimal configuration for Port 1 of the antenna booster element when integrated into two platforms with different form factors. A small platform measuring 50 × 50 mm^2^, equivalent to 0.11 λ at 698 MHz (Figure 3), and a larger platform measuring 142 × 60 mm^2^, equivalent to 0.33 λ at 698 MHz (Figure 4). The goal is to achieve optimal antenna multiband performance across frequencies 698–960 MHz (LFR) and 1710–2690 MHz (HFR).

Table 1 illustrates the relative bandwidth potential for both platforms obtained using a matching network synthesizer [36]. The results indicate that short-circuiting Port 1 offers better performance for the LFR, while leaving it open is preferable for the HFR. However, achieving optimal bandwidth for both regions simultaneously within the same element presents a challenge. Thus, can we have within the same element a behavior that obtains the bandwidth for LFR, as in the case of having a short circuit at Port 1, and, at the same time, the bandwidth at HFR, as in the case of an open circuit? A band-reject filter emerges as a viable strategy. This filter acts as a short circuit for the LFR frequencies, effectively attenuating (rejecting) the HFR signals by functioning as an open circuit for those frequencies. The following section will delve into the role of this filter in optimizing bandwidth across both frequency regions.

## 3. Design of the Filter

Previously, it has been indicated in Table 1 that the size of the antenna booster element significantly impacts the bandwidth distribution between the LFR and the HFR [37]. With a large antenna booster element, thanks to a short circuit at Port 1, the LFR experiences maximum bandwidth, while the HFR suffers from poor bandwidth. Conversely, when the antenna booster element is small, Port 1 is in an open circuit, and the HFR achieves maximum bandwidth at the expense of poor bandwidth in the LFR. Therefore, there is a need for high impedance in the HFR and low impedance in the LFR, leading to the proposal of a reject parallel LC filter between the stages of the antenna booster element at Port 1.

This LC filter must resonate at a frequency within the HFR to effectively attenuate HFR signals. However, there are multiple potential resonant frequency options, making it unclear which one is optimal. Equation (1) determines the resonant frequency (f_o_), which depends on the values of an inductance (L) and a capacitance (C). Given the variability in LC values and combinations, a new variable must be introduced.

The quality factor (Q) of a filter serves as a metric of its selectivity. A higher quality factor means a more precise or efficient filter, resulting in a narrower bandwidth around the center frequency. Equation (2) outlines the quality factor for an RLC circuit, where R represents resistance, C represents capacitance, and L represents inductance. As there is no resistance in the LC filter, subsequent analyses are conducted using the $\sqrt{C/L}$ ratio, referred to as Q_ratio_ (3).

To determine the ideal resonant frequency (f_o_) for rejection and the necessary level of selectivity for this filter (Q_ratio_), these equations are combined and examined through a heat map showcasing the potential bandwidth in each scenario.

(1) $$f_{o}=\frac{1}{2\pi \sqrt{LC}}$$

(2) $$Q=R\sqrt{\frac{C}{L}}$$

(3) $$Q_{ratio}=\sqrt{\frac{C}{L}}$$

The bandwidth potential of an antenna system can be assessed by estimating the achievable bandwidth considering the input impedance of the antenna system. Various methods exist for calculating this potential, and in this study, a matching network synthesizer is employed [38]. This approach involves designing impedance matching within the antenna system at a specific frequency, utilizing one or two lumped components.

**Figure 5:**
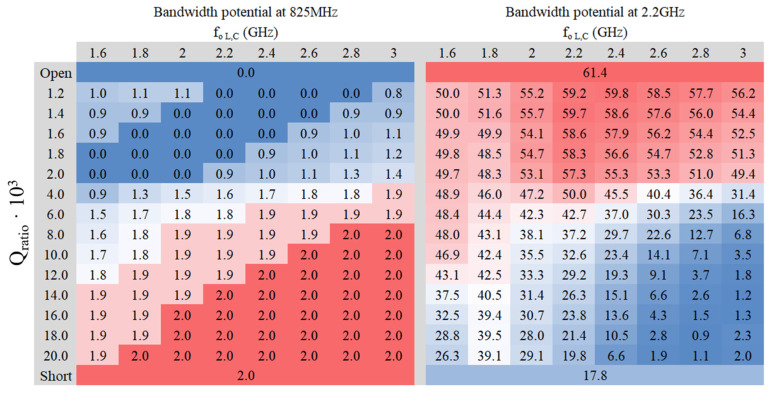
Heat maps of relative bandwidth (SWR = 3) in tant percent depending on the L and C values on a PCB of 50 mm × 50 mm at 825 MHz and 2.2 GHz.

**Figure 6:**
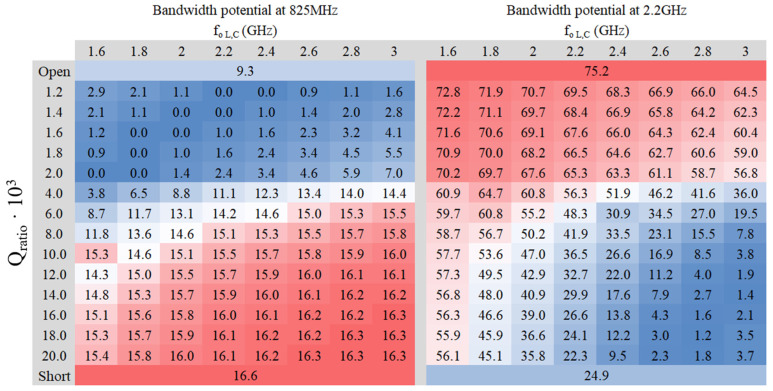
Heat maps of relative bandwidth (SWR = 3) in tant percent depending on the L and C values on a PCB of 142 mm × 60 mm at 825 MHz and 2.2 GHz.

A study of the relative bandwidth potential is conducted at a standing wave ratio (SWR) equal to three (S_11_ < −6 dB), considered as a reference for bandwidth computations in the context of IoT devices. The values of inductance (L) and capacitance (C) are adjusted based on the center frequency of the filter and the Q_ratio_. Two heat maps have been generated for each platform: one corresponding to the center frequency of the LFR and the other to the center frequency of the HFR (Figure 5 and Figure 6). Both figures were obtained using ideal components where R = 1 KΩ.

Analyzing the heat maps (Figure 5 and Figure 6) confirms that the maximum bandwidth potential occurs when Port 1 is in a short circuit in the LFR and an open circuit in the HFR. The filter needs to achieve a balance to ensure sufficient bandwidth for both frequency regions. Although the trend remains consistent in both PCBs 50 × 50 mm^2^ and 142 × 60 mm^2^, the absolute values differ significantly, particularly in the LFR, which is expected since bandwidth depends on the electrical size of the ground plane.

As outlined in Reference [39], the bandwidth can be doubled by incorporating custom-designed lumped components that adapt to the desired frequency. Traditionally, a 30% bandwidth is required to match frequencies from 698 MHz to 960 MHz. Nevertheless, following the methodology [39] reduces this requirement to only 15%. Similarly, 1710 MHz to 2690 MHz typically necessitates 44% of the bandwidth, yet employing the approach suggested reduces this requirement to 22%. However, the smaller 50 × 50 mm^2^ PCB cannot fully cover the entire LFR spectrum, whereas the larger 142 × 60 mm^2^ PCB can accommodate both frequency regions.

To validate this claim, numerical verification demonstrates that an adequate filter configuration can achieve a bandwidth surpassing the aforementioned 15% threshold, particularly in the LFR scenario. Even when doubling the bandwidth, the smaller 50 × 50 mm^2^ PCB remains insufficient to cover the entire frequency range of 698–960 MHz and 1710–2690 MHz. Consequently, a reconfigurable solution is recommended in such cases [40].

Turning attention to the larger PCB, which is capable of accommodating both frequency ranges, it is noticeable that the lower the Q_ratio_, the more bandwidth is available in the HFR. Conversely, a higher Q_ratio_ favors greater bandwidth in the LFR, aligning well with the findings from the previous study outlined in Table 1.

Examining Figure 6, it becomes apparent that at lower values of Q_ratio_·10^3^, the reactance of the filter in the LFR becomes highly inductive. For instance, at an f_o L,C_ of 1.2 GHz at 1.6 GHz, the reactance at 825 MHz is equal to 618 jΩ. Despite being high, it remains far from an open circuit. However, as the value of Qr_atio_·10^3^ decreases further, the inductance will increase, potentially leading to proximity to an open circuit. Delving deeper into Figure 6, two optimal zones emerge: one with a Q_ratio_·10^3^ value of 4 or 6, centered around frequencies of 2.2 GHz or 2.4 GHz, and another favorable zone with a Q_ratio_·10^3^ value of 20, centered at the frequency of 1.6 GHz. Thus, three potential filters in those zones are examined in Table 2 to determine the best approach.

**Figure 7:**
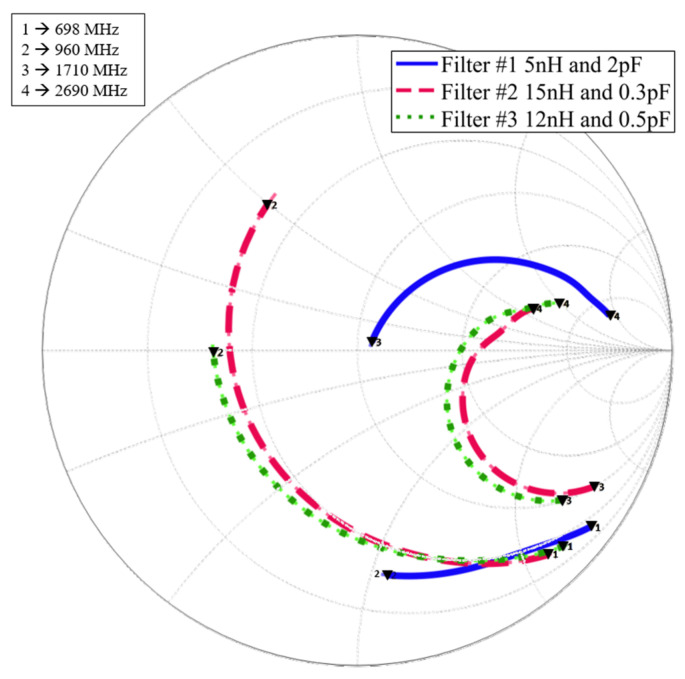
Smith chart of the LFR and HFR of the three filters.

To determine the optimal zone for filter design, Figure 7 presents a Smith chart illustrating the low-frequency range (LFR: 698–960 MHz) and high-frequency range (HFR: 1710–2690 MHz) of the three filters under consideration. Table 3 provides the real and imaginary parts of the impedances within the LFR and HFR for each filter.

The data indicates that the real part of the impedance remains relatively stable across all filters within the LFR. However, within the HFR, particularly in Filter #1, both the real and imaginary parts of the impedance exhibit greater variability. This variability suggests potentially poorer matching to a 50 Ω system impedance.

A secondary analysis was conducted to investigate whether deviating from the 50 Ω impedance leads to poorer matching. Equation (4) demonstrates the quality factor of a resonant filter, while Equation (5) outlines the method for determining bandwidth.

(4) $$Q_{a}\left(\omega \right)=\frac{\omega }{2R(\omega )}\;\sqrt{{\left[\frac{dR(\omega )}{d\omega }\right]}^{2}+{\left[\frac{dX(\omega )}{d\omega }+\left|\frac{X(\omega )}{\omega }\right|\right]}^{2}\;}$$

(5) $$BW=\frac{f_{2}-f_{1}}{f_{o}}=\frac{SWR-1}{Q_{a}\sqrt{SWR}}$$

**Figure 8:**
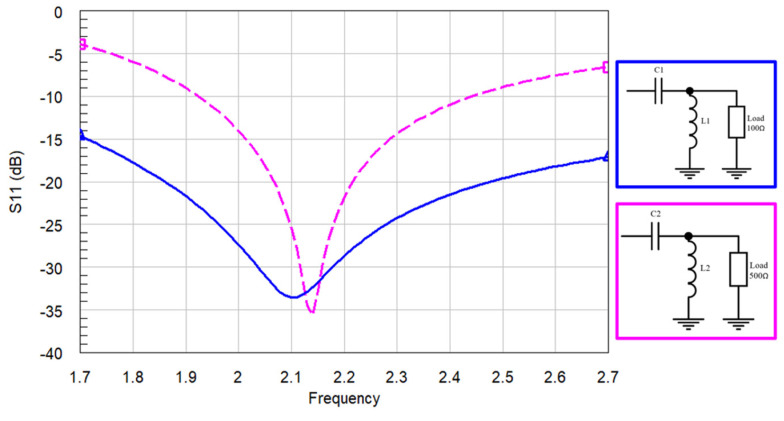
Loads of 100 Ω and 500 Ω matched at 50 Ω with an LC in HFR.

Ideally, matching a 100 Ω or 500 Ω load to 50 Ω should result in infinite bandwidth. However, Figure 8 shows that the 100 Ω load achieves a wider bandwidth compared to the 500 Ω load when examining the performance of Filter #1. This observation suggests that Filter #1 might not perform as well as Filters #2 and #3 in terms of adapting to the 50 Ω system impedance.

**Figure 9:**
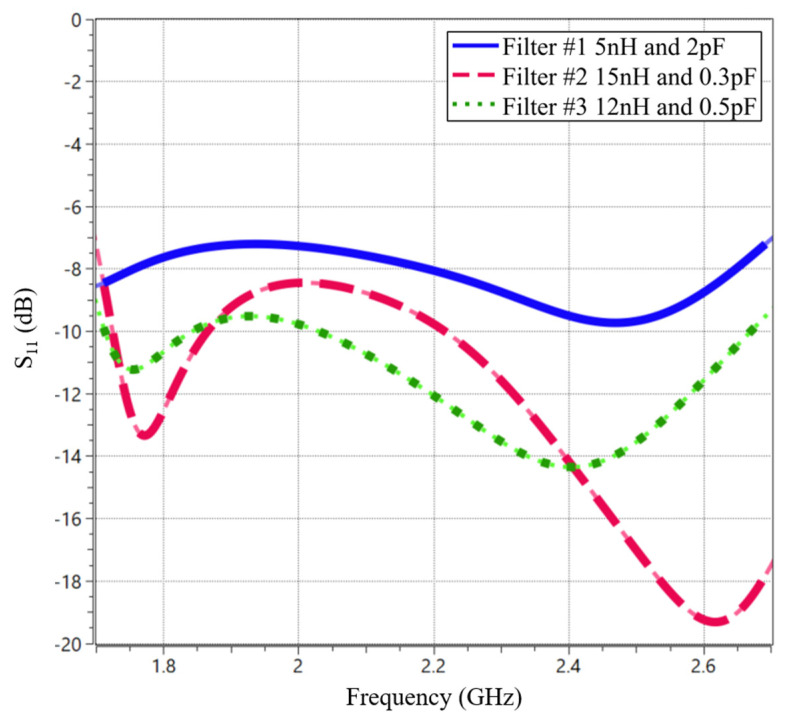
Simulated S_11_ of the filters, adding a matching network with two ideal components LC to adapt the HFR band.

To confirm this hypothesis, simulations are conducted. All three filters are adapted to the high-frequency range (1710–2690 MHz) using ideal LC components. As shown in (Figure 9), Filters #2 and #3 exhibit a deeper S_11_ compared to Filter #1.

## 4. Electromagnetic Simulations

Continuing the filter design process, three simulations are conducted, one for each proposed filter (Filters #1, #2, and #3). To adapt the design for both LFR and HFR, a matching network is designed and placed at the RF1 port, with a maximum of six lumped components. These simulations are conducted using a matching network synthesizer [36] to identify the optimal matching network and an electromagnetic simulator software to analyze the S parameters and total efficiency. Simulations are performed using high-Q components, which incorporate the inherent losses of real components.

**Figure 10:**
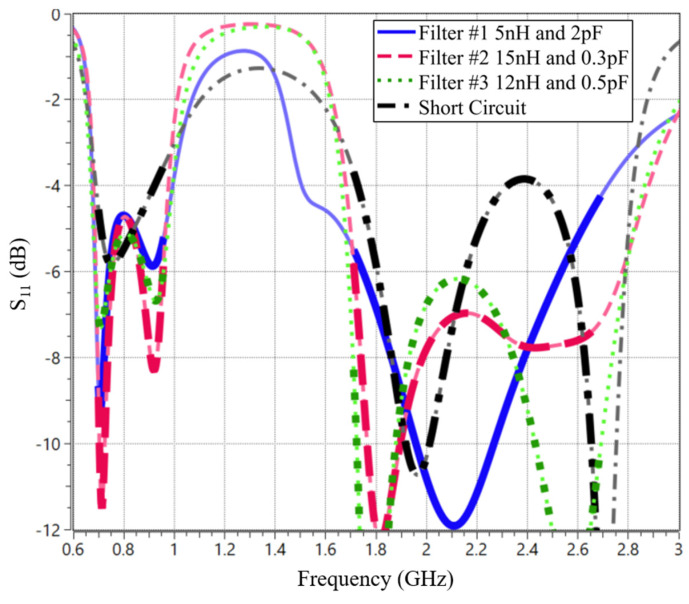
Simulated S_11_ of each filter with their matching network to adapt LFR and HFR.

An examination of the S_11_ parameters (Figure 10) reveals that Filter #1 exhibits poorer matching compared to Filters #2 and #3. Nonetheless, all three filters outperform the scenario where Port 1 is short-circuited.

Furthermore, Table 4 summarizes the average simulated total efficiency achieved by each design. This includes the chosen filter and the optimized matching network, designed to handle both LFR and HFR at the RF1 port. The matching networks are limited to a maximum of six lumped components. As expected, the Port 1 short-circuited represents the worst-case scenario, exhibiting the lowest average efficiency in both LFR and HFR.

Upon comparison between the filters, it is observed that all three exhibit similar efficiencies despite differences in their performance in LFR and HFR. Filter #1 performs better in HFR, whereas Filters #2 and #3 excel in LFR. In conclusion, among the two optimal zones, the most suitable for multiband applications is characterized by a value between 4 and 6 for Q_ratio_·10^3^, with a centered frequency spanning from 2.2 GHz to 2.4 GHz.

**Figure 11:**
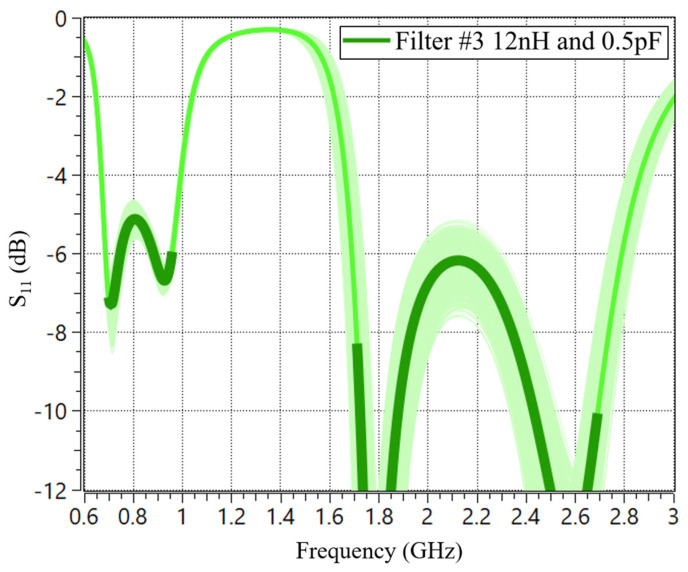
Tolerance analysis of Filter #3, 12 nH, and 0.5 pF, with the matching network of 6 components in RF1.

Before physically implementing two solutions of Filters #2 and #3, a tolerance analysis is conducted involving 1000 circuit evaluations to ensure the robustness of the matching networks. Tight tolerance is considered (2%), which is available in most commercial components (Figure 11). As observed, both solutions show resilience concerning component tolerance.

## 5. Experimental Validation

**Figure 12:**
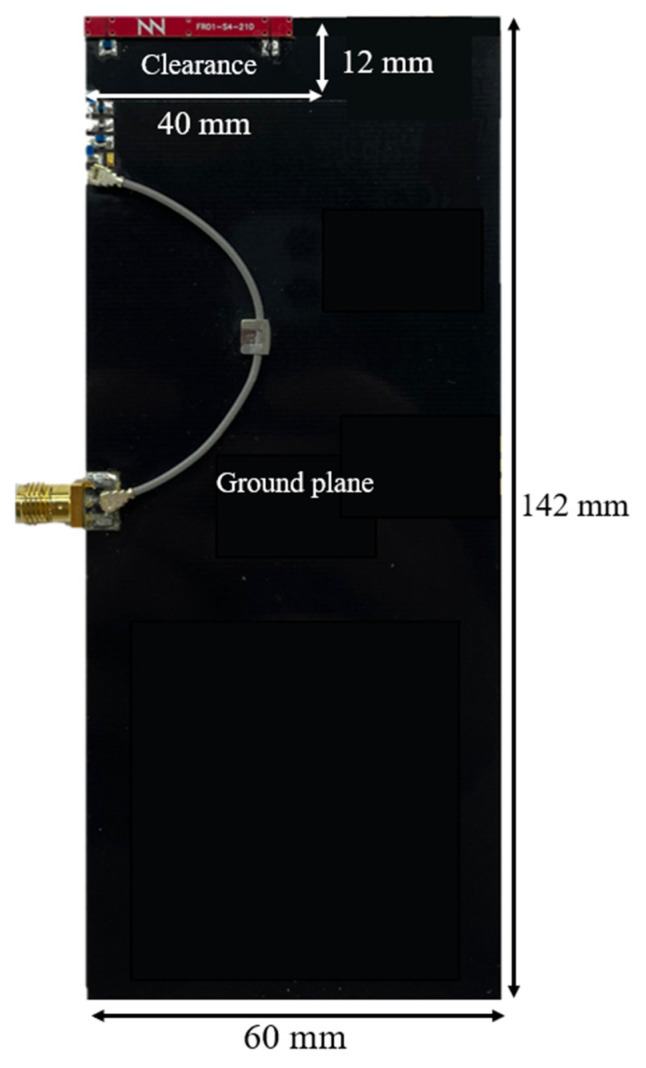
Physical implementation of a PCB of 142 mm by 60 mm with FR4 substrate of 1 mm thick (ε_r_ = 4.15, tanδ = 0.02) with a clearance area of 12 mm by 40 mm within a 30 mm × 3 mm × 1 mm (height) antenna booster element.

To validate the simulation results, a physical prototype of the 142 mm × 60 mm PCB, with a 12 mm × 40 mm clearance area, is implemented using an FR4 substrate 1 mm thick, with a dielectric constant (ε_r_) of 4.15 and a loss tangent (tanδ) of 0.02 (Figure 12).

**Figure 13:**
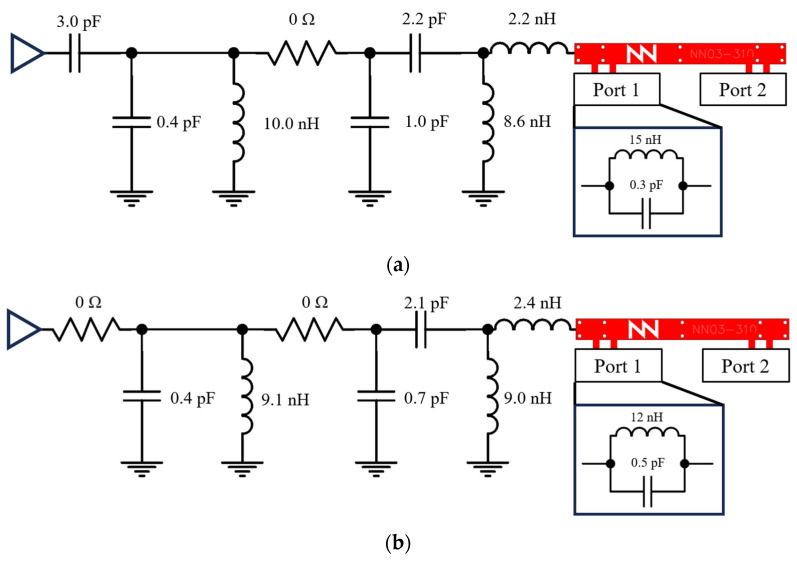
Matching networks to match 698–960 MHz and 1710–2690 MHz with a platform of 142 × 60 mm^2^: (**a**) with Filter #2 and (**b**) with Filter #3, both with Port 2 in a short circuit.

Both filters identified as optimal, #2 (with 15 nH and 0.3 pF) and #3 (with 12 nH and 0.5 pF), are implemented and tested. The corresponding matching networks designed to adapt from 698 MHz to 960 MHz and from 1710 MHz to 2690 MHz are depicted (Figure 13). As previously discussed, Port 2 can be used for fine-tuning. To explore this option, tests are conducted with Port 2 in both short circuit and open circuit. These marching networks are designed with high-Q components, ensuring performance consistency across the frequency ranges specified. The worst-case scenario in terms of Q-factor is detailed in Table 5.

**Figure 14:**
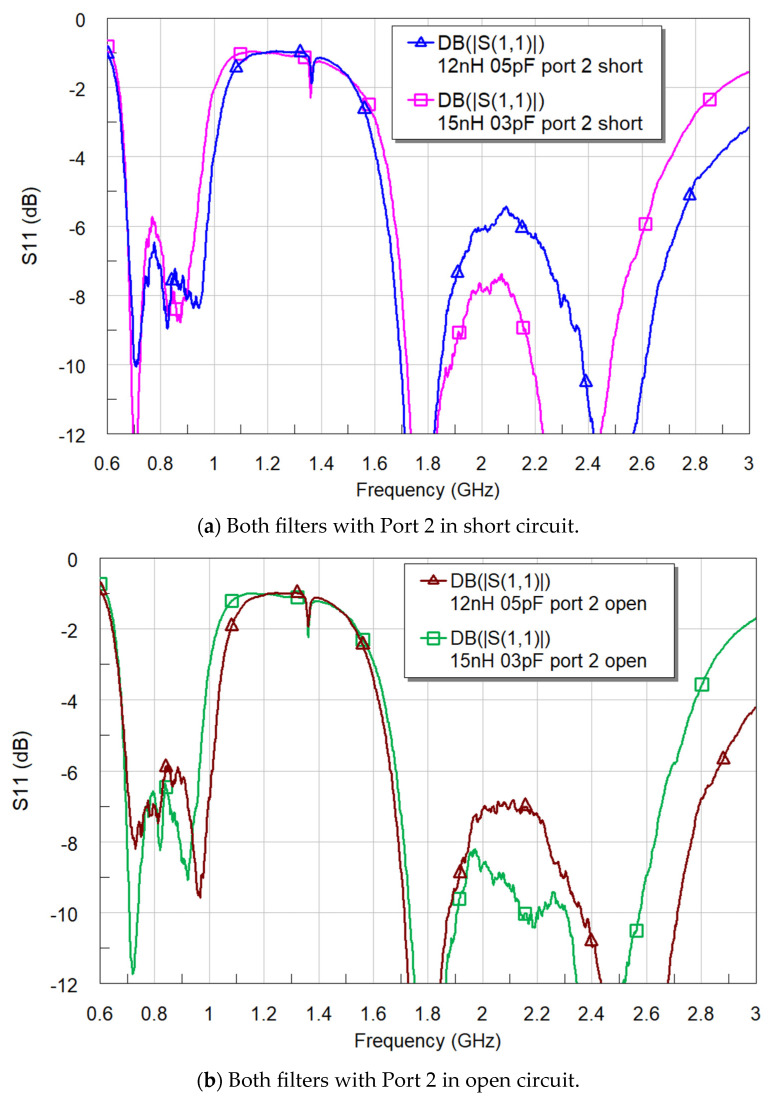
S_11_ with both filters and the matching networks shown in (Figure 12).

The S_11_ results reveal that both filters exhibit satisfactory matching in both LFR and HFR (Figure 14). When comparing the filters, Filter #2 (15 nH and 0.3 pF) with Port 2 configured as an open circuit demonstrates the best performance in both LFR and HFR. It is worth noting that both filters achieve the target of SWR < 3 (S_11_ < −6 dB), indicating overall good performance across the frequency spectrum.

**Figure 15:**
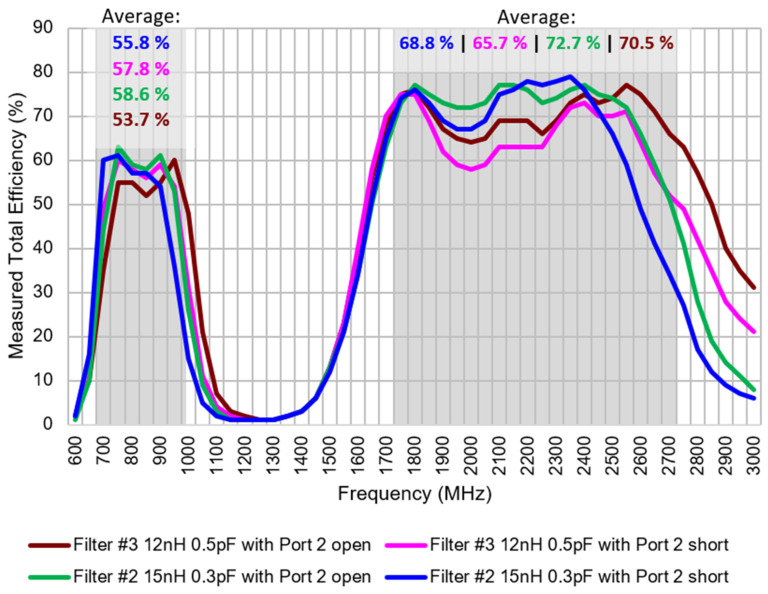
Measured total efficiency.

**Figure 16:**
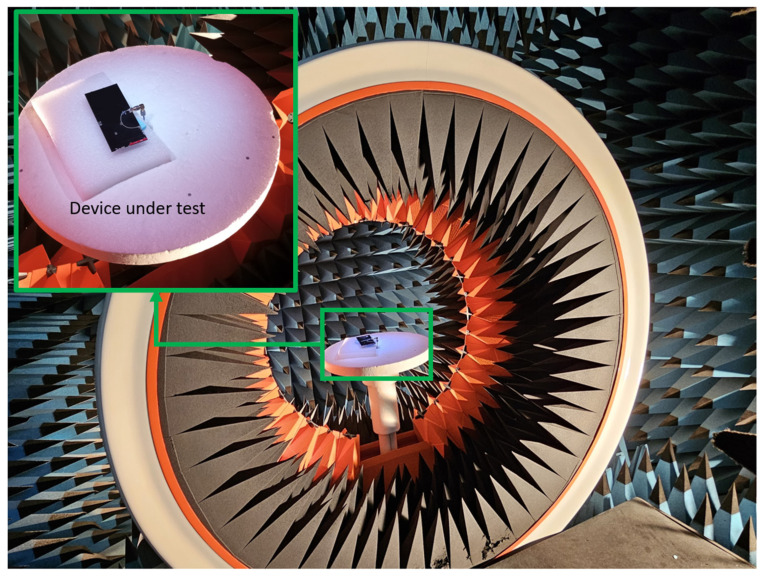
Measurement setup for total efficiency in the anechoic chamber.

Total efficiency (η_t_) is measured in an anechoic chamber at Ignion lab(Star-Lab 18 from MVG) (Figure 15 and Figure 16). It includes losses of the PCB, the antenna element, the matching network, and the micro-coaxial line. The analysis reveals a comparable performance between both filters. Specifically, examination of the S parameters indicates that the average total efficiency in the LFR for Filter #3 slightly surpasses that of Filter #2. Conversely, when considering the average total efficiency in the HFR, Filter #2 exhibits a slight advantage over Filter #3. However, these differences are negligible, affirming the effective functionality of both filters.

For certain narrowband-IoT (NB-IoT) standards concerning total radiated power (TRP) in free space [41], the antenna system needs to achieve specific power levels: 18 dBm in the LFR (698–960 MHz) and 20 dBm in the HFR (1710–2690 MHz). Considering an estimated RF module output power of 23 dBm, the design targets a minimum total efficiency of 32% in the LFR and 50% in the HFR. The efficiency measurements confirm that both filters surpass these minimum requirements to operate correctly.

**Figure 17:**
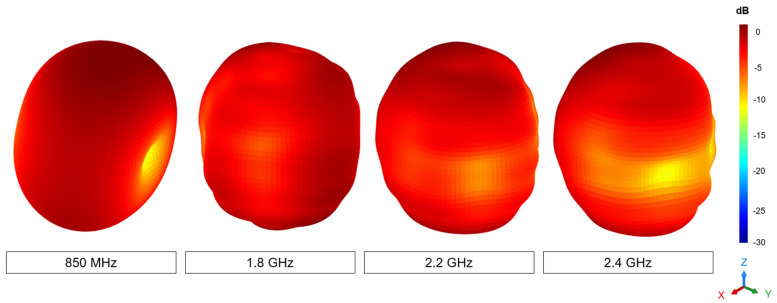
Measured 3D radiation pattern showing the antenna realized gain at 850 MHz, 1.8 GHz, 2.2 GHz, and 2.4 GHz of Filter #2 with Port 2 in open circuit.

An examination of the radiation pattern of the optimal antenna configuration, featuring Filter #2 (15 nH and 0.3 pF) in Port 1, a matching network in RF Port 1, and an open circuit in Port 2, reveals a quasi-isotropic pattern with a directivity of approximately 3 dBi (Figure 17). Regarding realized gain, this antenna system attains 0.7 dBi at 850 MHz, 2.1 dBi at 1.8 GHz, 2.4 dBi at 2.2 GHz, and 2.6 dBi at 2.4 GHz.

This characteristic proves valuable in IoT devices, as it accommodates random signal directions and device orientations, which are common occurrences in IoT communication scenarios. Consequently, it is well-suited for scenarios where omnidirectional signal reception or transmission is more important than directional focus, such as in the context of IoT devices.

While directivity remains around 3 dBi in all cases, the gain fluctuates due to changes in efficiency. For instance, in the optimal scenario of Filter #2 with Port 2 open-circuited, the efficiency at 850 MHz is −2.37 dB, and at 2.2 GHz, it is −1.19 dB, which accounts for the variation in gain.

## 6. Discussion

To verify the performance of this antenna, a comparative analysis with existing literature is conducted (Table 6). This comparison includes other studies that use the same frequency range: the LFR from 698 MHz to 960 MHz and the HFR from 1710 MHz to 2690 MHz. The table presents the antenna dimensions along with the clearance area, the size of the PCB, and the average efficiency in both LFR and HFR. The antenna booster element not only presents the smallest height (1 mm) compared to the other elements but also a smaller volume of only 90 mm^3^, more than ten times smaller than the smallest antenna element in the list. This helps to integrate the solution into compact and low-profile wireless devices.

Regarding total efficiency, the proposed solution is only smaller compared to [8,9] for LFR at the expense of a much larger antenna size. For example, the antenna length in [8] is 80 mm, which is difficult to integrate into IoT devices featuring widths less than 60 mm. The same occurs with [9]; the antenna length is 55 mm, which makes it prohibitive for devices featuring a width of less than 50 mm, in addition to its height of 8 mm compared to only 1 mm for the antenna booster element. Note that the present antenna booster element can be integrated into such narrow devices due to the short length of only 30 mm [42]. At HFR, efficiency is only better for [8], which again includes a large antenna of 55 mm and a height of 8 mm. For the other antennas, the antenna booster element presents better efficiency, a smaller size, and a lower height.

**Figure 18:**
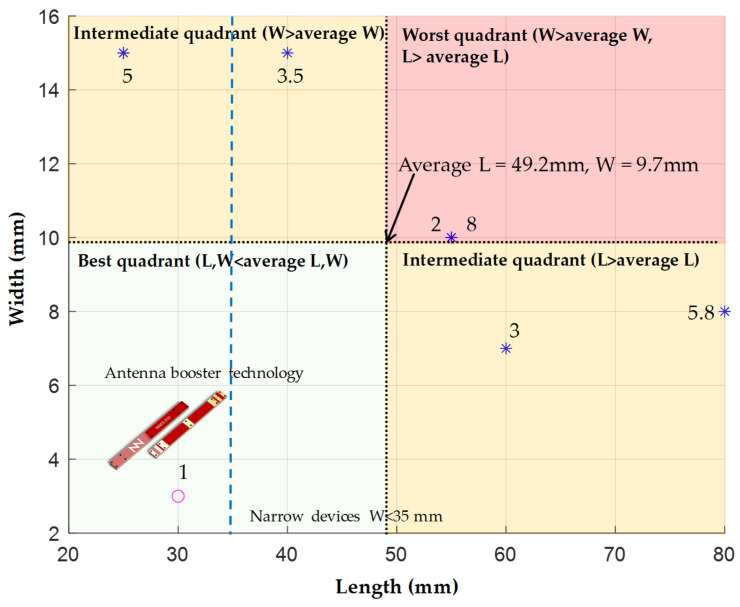
Classification of the dimensions of the antenna compared to previous work (Table 6). Note that the 30 mm × 3 mm × 1 mm antenna booster is in the best quadrant where both L and W are less than the average L and W. The number associated with each marker is the antenna height.

A comparison of the antenna dimensions of the designs from Table 6 is shown in (Figure 18). The average antenna size based on the length and width of all entries is calculated. This average is used to define four quadrants: best, worst, and two intermediate categories with either below-average length and above-average width or vice versa. The results are that this new research presents the only antenna design located within the best quadrant. Additionally, it has the lowest height among all the compared designs, simplifying integration as it remains lower than the height of a typical RF module mounted on a PCB and, thus, enabling slim wireless devices with narrow widths (width ≤ 35 mm) [43].

## 7. Conclusions

This study explored achieving multiband performance (698–960 MHz and 1710–2690 MHz) for an antenna booster element. By analyzing the antenna’s ports, the need for a band-reject filter at Port 1 is identified to optimize operation across both frequency ranges. An extensive analysis of bandwidth potential revealed two promising zones for filter design. Subsequently, three potential filter configurations are evaluated using electromagnetic simulations. This analysis identified the most suitable design for multiband applications with a Q_ratio_·10^3^ value between 4 and 6 and a centered frequency ranging from 2.2 GHz to 2.4 GHz. Experimental validation confirmed comparable performance between the two filter implementations. Both cases achieved the desired S_11_ parameter (<−6 dB), indicating good matching, and delivered similar average total efficiency. The radiation pattern analysis revealed a quasi-isotropic characteristic with a directivity of approximately 3 dBi. This omnidirectional radiation pattern is particularly advantageous for IoT devices, as it ensures reliable communication regardless of signal direction or device orientation.

## Figures and Tables

**Table 1:** Bandwidth Potential (SWR = 3).

Port 1	Platform	698 MHz to 960 MHz	1710 MHz to 2690 MHz
Short circuit	50 mm × 50 mm	2.0%	17.8%
	142 mm × 60 mm	16.6%	24.9%
Open circuit	50 mm × 50 mm	~0%	61.4%
	142 mm × 60 mm	9.3%	75.2%

**Table 2:** Filters in Study.

	C (pF)	f_o_ (GHz)	Q_ratio_·10^3^
5	2	1.6	20
12	0.5	2.05	6.45
15	0.3	2.37	4.47

**Table 3:** Impedance in LFR (696–960 MHz) and HFR (1710–2690 MHz) for Each Filter.

Filter #1 5 nH and 2 pF	LFR	18 ≤ Re{Z} ≤ 19
		−148 ≤ Im{Z} ≤ −54
	HFR	55 ≤ Re{Z} ≤ 213
		3 ≤ Im{Z} ≤ 337
Filter #2 15 nH and 0.3 pF	LFR	Re{Z} = 19
		−113 ≤ Im{Z} ≤ 25
	HFR	50 ≤ Re{Z} ≤ 156
		−174 ≤ Im{Z} ≤ 62
Filter #3 12 nH and 0.5 pF	LFR	Re{Z} = 19
		−123 ≤ Im{Z} ≤ 0
	HFR	50 ≤ Re{Z} ≤ 187
		−136 ≤ Im{Z} ≤ 99

**Table 4:** Average Simulated Total Efficiency Matching with Six or less Lumped Components.

Port 1	698 MHz to 960 MHz	1710 MHz to 2690 MHz
Short circuit	56.9%	65.1%
Filter #1 5 nH and 2 pF	58.9%	79.0%
Filter #2 15 nH and 0.3 pF	61.8%	75.1%
Filter #3 12 nH and 0.5 pF	60.9%	78.8%

**Table 5:** Worst Quality Factor Q in the Frequency Range of Interest (698–2690 MHz) of the Components in Figure 12.

15 nH	12 nH	10 nH	9.1 nH	9.0 nH	8.6 nH	2.4 nH	2.2 nH
79	80	84	80	80	79	60	73
**3.0 pF**	**2.2 pF**	**2.1 pF**	**1.0 pF**	**0.7 pF**	**0.5 pF**	**0.4 pF**	**0.3 pF**
87	97	103	223	263	343	405	459

**Table 6:** Comparison with Other Works with antennas covering 698–960 MHz and 1710–2690 MHz. Antenna volume is considered the smallest parallelepiped, including the antenna element.

Reference	Dimensions of the Antenna (mm^3^)	Antenna Volume (mm^3^)	Non-Ground Portion—Clearance Area (mm^2^)	PCB Size (mm^2^)	Measured Average Efficiency LFR (%)	Measured Average Efficiency HFR (%)
[5]	40 × 15 × 3.5	2100	40 × 15	120 × 60	47	66
[6]	25 × 15 × 5	1875	25 × 15	120 × 60	43	55
[7]	55 × 10 × 2	1100	55 × 10	120 × 55	53.5	57.8
[8]	55 × 10 × 8	4400	55 x10	115 × 55	75	82
[9]	80 × 8 × 5.8	3712	80 × 8	140 × 80	83	61
[10]	60 × 7 × 3	1260	60 × 7	120 × 60	NA	NA
**This Work**	**30 × 3 × 1**	**90**	**40 × 12**	**142 × 60**	**58.6**	**72.7**

## Data Availability

Data are contained within the article.
